# HVPG signature: A prognostic and predictive tool in hepatocellular carcinoma

**DOI:** 10.18632/oncotarget.11558

**Published:** 2016-08-23

**Authors:** Xiaolong Qi, Xin Zhang, Zhijia Li, Jialiang Hui, Yi Xiang, Jinjun Chen, Jianbo Zhao, Jing Li, Fu-Zhen Qi, Yong Xu

**Affiliations:** ^1^ Department of General Surgery, Nanfang Hospital, Southern Medical University, Guangzhou, China; ^2^ Department of Radiology, the Fourth People's Hospital of Huai'an, Huai'an, China; ^3^ Department of Infectious Diseases, Nanfang Hospital, Southern Medical University, Guangzhou, China; ^4^ Department of Interventional Radiology, Nanfang Hospital, Southern Medical University, Guangzhou, China; ^5^ Department of Gastroenterology, Tongji Hospital of Tongji University, Shanghai, China; ^6^ Department of Hepatopancreatobiliary Surgery, Huai'an First People's Hospital, Nanjing Medical University, Huai'an, China; ^7^ Department of Nephrology, The Affiliated Huai'an Hospital of Xuzhou Medical University and The Second People's Hospital of Huai'an, Huai'an, China

**Keywords:** hepatic venous pressure gradient, hepatocellular carcinoma, prognosis, prediction, hepatic resection

## Abstract

Hepatic venous pressure gradient (HVPG) measurement provides independent prognostic value in patients with cirrhosis, and the prognostic and predictive role of HVPG in hepatocellular carcinoma (HCC) also has been explored. The management of HCC is limited to the European Association for the Study of the Liver (EASL) and American Association for the Study of Liver Diseases (AASLD) guidelines that consider that HVPG≥10 mmHg to be a contraindication for hepatic resection (HR), otherwise other treatment modalities are recommended. Current studies show that a raised HVPG diagnosed directly or indirectly leads to a negative prognosis of patients with HCC and cirrhosis, but HVPG greater than 10 mmHg should not be regarded as an absolute contraindication for HR. Selecting direct or surrogate measurement of HVPG is still under debate. Only several studies reported the impact of HVPG in negative prognosis of HCC patients after liver transplantation (LT) and the value of HVPG in the prediction of HCC development, which need to be further validated.

## INTRODUCTION

Hepatocellular carcinoma (HCC) is a major health problem worldwide and the main cause leading to death in patients with cirrhosis [1]. Currently, the treatment modalities of HCC include hepatic resection (HR), liver transplantation (LT) and so on, and each of them has its own indications [2]. The European Association for the Study of the Liver (EASL) and American Association for the Study of Liver Diseases (AASLD) guidelines for the management of HCC consider a hepatic venous pressure gradient (HVPG) ≥10 mmHg to be a contraindication for HR [2, 3] due to the higher risk of postoperative liver decompensation based on the reports of Barcelona Clinic Liver Cancer (BCLC) group [4, 5]. Accordingly, HR should be considered the first treatment option for HCC patients with normal bilirubin and either platelet count≥100,000 /mm^3^ or HVPG <10 mmHg in BCLC stage 0 with a single nodule (size≤2 cm) of HCC, otherwise other treatment strategies, such as LT and radiofrequency ablation (RFA), are recommended [2, 3]. The indications of LT are HCC patients with increased bilirubin and platelet count ≤100,000 /mm^3^ in BCLC stage A-C with single or 3 nodules nodule≤3 cm [2].

HVPG measurement helps to provide prognostic information on survival and the risk of hepatic decompensation in patients with cirrhosis or HCC [6, 7] and is the gold standard for the estimation of the presence and severity of portal hypertension (PHT) which is defined by an increase in portal pressure above the normal range (≤5 mm Hg), as measured by HVPG [6]. It is defined as clinically significant portal hypertension (CSPH) when the value of HVPG exceeds 10 mmHg. The occurrence of CSPH is a special threshold because it increases the risks of developing esophageal varices (EV) and HCC [6, 8] as well as hepatic decompensation in the evolution of cirrhosis [6] and after HR for HCC [4–7]. The value of HVPG is the difference between the wedged hepatic venous pressure and the free hepatic venous pressure [6]. Given that the direct transjugular measurement of HVPG is invasive and available only in specialized centers [2, 6], EASL recommends surrogate measures of CSPH in practice including the presence of EV or platelet count < 100000/mm^3^ associated with splenomegaly [2]. In this report, we review the predictive and prognostic value of HVPG in HCC and also the debates about different measurements of CSPH and current guidelines for HCC.

## THE VALUE OF HVPG IN HCC PROGNOSIS AFTER HR

### The clinical outcomes of HR in patients with HCC and CSPH

In cirrhotic patients, it is acknowledged that preoperative HVPG measurement provides independent prognostic information on the risk of decompensation and survival [6, 9].A decrease in HVPG of at least 20% or to less than 12 mmHg is significantly associated with a reduction in variceal rebleeding and defines patients as responders in response to drug therapy [6, 9].

It is since 1990s that researchers have started to explore whether HVPG has or not have prognostic value in HCC patients. Two previous seminal BCLC studies in 1996 and 1999 showed that in HCC patients with compensated cirrhosis, the absence of CSPH and normal bilirubin promised a good prognosis after HR [5] with a 5-year survival > 70% [4]. By contrast, CSPH increased the risk of postoperative clinical decompensation and the survival of patients with CSPH was significantly reduced to 50-60% [4, 5], which contributed to the guidelines from EASL and AASLD [2, 3]. However, no additional evidence supported these results for more than one decade [7].

Currently, the preoperative elevated HVPG directly diagnosed has been confirmed to correlate significantly with postoperative liver dysfunction [10, 11], clinical decompensation [12, 13] and shorter survival [10] and to be used to stratify the risk of post-hepatectomy liver failure (PHLF) in patients with HCC and cirrhosis [12]. And HCC patients with CSPH assessed by surrogate measures also have lower overall survival (OS) [7, 14] and higher recurrence rates [14]. A meta-analysis demonstrated that the presence of CSPH evaluated by direct or surrogate measurements was significantly associated with the higher risk of 3- and 5-year mortality and of hepatic decompensation after HR for HCC [7]. But these results were not regarded as convincing evidence mainly due to the differences of definitions or evaluations of CSPH, BCLC stage and endpoints in studies included [15, 16].

On the contrary, studies have reported that there were no differences between surrogate CSPH and non-CSPH patients with HCC and compensated cirrhosis after HR in recurrence-free survival (RFS) [17] and OS [12, 17, 18]. *He W* and colleagues reported that before propensity score matching, CSPH was associated with higher risks of postoperative complication and liver decompensation that were similar after propensity score matching [17]. The different results may be explained by eliminated covariates, minimized their confounding effect and made demographic and clinical characteristics similar [17]. There is only one study reporting CSPH diagnosed directly had no impact on 3-, 5-year survival but declaring that the result may result from small numbers enrolled and relatively short follow-up [12].

Since most studies support that CSPH measured directly or indirectly is negative prognostic factor for patients with HCC and cirrhosis, it is necessary for clinicians to control the raised HVPG when managing HCC (Table 1).

**Table 1:** Association between elevated HVPG and prognosis of HCC with cirrhosis after hepatic resection

Study	Inclusion period	Measurements of HVPG	No. of surgical cases	No. of elevated HVPG	Main Endpoints	Conclusions
Boleslawski, 2012 [10]	2007-2009	directly	40	≥10 mmHg 18 (45.0%)	Postoperative liver dysfunction	A raised HVPG was associated with postoperative liver dysfunction and 90-day mortality.
Stremitzer, 2011 [11]	2000-2009	directly	35	≥5mmHg 14 (40.0%)	Postoperative complications and death	HVPG exceeding 5 mmHg was associated with worse liver fibrosis, higher rates of postoperative liver dysfunction and ascites and a longer hospital stay.
Cucchetti, 2016 [12]	2009-2014	directly	70	≥10 mmHg 34 (48.6%)	Post-hepatectomy liver failure defined by the International Study Group of Liver Surgery, 90 day mortality, Detailed clinical evaluation after 3 months	HVPG can be used to stratify the risk of post-hepatectomy liver failure. CSPH was associated with a higher risk of ascetic decompensation. But there was no difference in 1- and 3- survival rates after resection between CSPH group and non-CSPH group.
Ripoll, 2007 [13]	1993-1999	directly	213	≥10 mmHg 134 (62.9%)	Development of clinical decompensation	HVPG can predict clinical decompensation in patients with compensated cirrhosis. Patients without CSPH have a 90% probability of not developing clinical decompensation in a median follow-up of 4 years.
Ishizawa, 2008 [14]	1994-2004	the presence of EV and/or PC of 100,000/L associated with splenomegaly	386	≥10 mmHg 136 (35.2%)	Recurrence, 3-year/5-year mortality	Long-term outcomes were poorer in CSPH group than in the no-CSPH group among patients with Child-Pugh class A cirrhosis but did not differ in two groups among patients with Child-Pugh class B cirrhosis
He, 2015 [17]	2003-2008	if two or more of the criteria were met: 1) PC < 100 × 10^9^/l and/or white blood cell count < 4 ×10^9^/l three times in succession, 2) Splenomegaly, 3) Portal vein width > 14 mm or spleen vein width > 10 mm via ultrasound, and 4) EV.	209	≥10 mmHg 102 (48.8%)	Recurrence, Liver decompensation, 5-year mortality	Before propensity score matching, CSPH patients had higher rates of postoperative complication and liver decompensation with similar rates of recurrence-free survival and overall survival. However, after propensity score matching, revealed similar rates of postoperative complication, liver decompensation, recurrence-free survival and overall survival.
Giannini, 2013 [18]	1987-2008	the presence of either EV or gastric varices, portal hypertensive gastropathy, or PC < 100 × 10^9^/l associated with splenomegaly	152	≥10 mmHg 68 (44.7%)	Death or until December 2008	Presence of CSPH has no influence on survival of HCC patients with well-compensated cirrhosis.

CSPH= clinically significant portal hypertension, EV= esophageal varices, HCC= hepatocellular carcinoma, HVPG= hepatic venous pressure gradient, PC=Platelet count.

### CSPH should not be regarded as an absolute contraindication to HR

The EASL and AASLD guidelines for the management of HCC consider CSPH to be a contraindication for HR [2, 3] due to the higher risk of postoperative liver decompensation based on the reports of BCLC group [4, 5]. However, there is no consensus regarding how CSPH should be evaluated in the decision-making process, but most researches hold the view that CSPH should not be considered an absolute contraindication to HR [12, 15, 16, 19].

Firstly, although the present studies have confirmed that CSPH was associated with a higher risk of clinical decompensation and shorter survival after HR [10, 12], the strict application of CSPH excludes approximately one-quarter of the HCC patients who would benefit from HR [12]. Secondly, CSPH is a marker of a more advanced evolutionary stage of cirrhosis [6, 7], which is linked to worse prognosis, and it is necessary for studies to regard the preoperative HVPG with a longer longitudinal assessment [12]. Thirdly, the extent of resection and tumor number are also the risk factors of clinical outcomes after HR [14, 20, 21], which may be ignored when designing proposals [17]. And the small numbers enrolled and short follow-up of those studies are also factors influencing to make robust conclusions that HCC patients with CSPH should be absolutely excluded from HR [12]. Fourthly, the improvements in surgical and perioperative care can lower the mortality associated with HR [15, 21, 22]. Last but not least, the current guidelines are based on the principle of selecting candidates achieving the best results after HR [2, 3], but to a particular patient, even if he/she may not be ideal candidate for HR, HR may still be the best choice of all treatment strategies [23]. It is very necessary to point out that these guidelines mentioned above may not apply generally in Asia.

If the patients had early-stage HCC or had undergone relatively limited hepatectomy, short- and long-term OS and recurrence rates were similar between CSPH and non-CSPH patients [19] and HR may benefit HCC patients with CSPH [19, 23]. Besides, *Boleslawski et al* argued that since direct HVPG cannot agree with surrogate measures of CSPH on the impact of clinical outcomes, it was still questionable to exclude HCC patients with CSPH assessed by surrogate measurements from HR [10].

In general, HVPG (regardless of direct or surrogate measurements) reflects liver function to some extent, but the presence of CSPH should not be regarded as an absolute contraindication to HR. It is necessary to take various factors (e.g. surgical techniques, preoperative care and laparoscopic / open HR) into consideration before selecting treatment strategies.

### Surrogate measurements of HVPG

Direct measurement of HVPG is not routinely applied because of its invasion and availability only in specialized centers, EASL recommends surrogate measures of CSPH in practice including the presence of EV or platelet count < 100000/mm^3^ associated with splenomegaly [2, 6, 23]. Most studies researching the association between HVPG and clinical outcomes in HCC patients with cirrhosis have various surrogate measurements of CSPH (Table 1), such as imaging examinations and hepatofugal portal flow, except those from EASL [2, 7, 15]. Few studies have specifically addressed whether these indirect criteria are accurate enough to replace the direct measurement of HVPG since HVPG measurement is regarded as the gold standard for estimating the presence and severity of PHT [7, 12, 14].

These are two studies that compared respectively the strength between direct and surrogate criteria of CSPH and clinical prognosis [7, 10]. One research published in 2012 by *Boleslawski* and *colleagues* [10] described a series of 40 patients with HCC and liver cirrhosis, in whom a preoperative raised HVPG was proved to be independently associated with higher risks of postoperative liver dysfunction and 90-day mortality after HR, whereas indirect criteria of CSPH (EV and/or splenomegaly associated with thrombocytopenia) were not, which suggested that preoperative HVPG should be measured directly whenever possible in HCC patients with cirrhosis [10]. Another study, the meta-analysis [7] mentioned previously, showed that the strength of the relationship between CSPH evaluated by direct measures and clinical outcomes after HR was greater, which was particularly evident in the assessment of postoperative clinical decompensation. Furthermore, direct HVPG was also suggested to be used to evaluate CSPH except for patients with EV which relied on the presence of CSPH [7, 9]. However, this study was based on a wide variety of endpoints and the results are still under debate [24].

Actually, patients who have no varices or splenomegaly associated with thrombocytopenia but cannot be confidently ruled out CSPH are present in up to 40% [7, 25, 26], and there may be selection bias when taking surrogate measurements of CSPH. Moreover, it has been confirmed that EV only forms in patients with cirrhosis and HVPG value of at least 10 mmHg which is the pathophysiological basis of the formation of EV, so the presence of EV can confidently confirm CSPH [6, 9]. On the other hand, not all conditions of CSPH diagnosed by various surrogate measurements are actually equal to or exceed the cutoff of 10 mmHg [10]. By contrast, direct HVPG is more precise to figure out even small increases of portal pressure, which may be meaningful to impact on the clinical outcomes [6, 7].

As already reported, liver stiffness (LS) measured by transient elastography has been shown to correlate strongly with HVPG and appeared to be the best candidate as a noninvasive surrogate marker for HVPG [27, 28], and about half of patients with potentially resectable HCC can be classified as having or not CSPH by LS [25]. Once HVPG values exceed 10 to 12 mm Hg, which are the threshold of CSPH, HVPG becomes independent from LS [27, 28]. Besides, LS measurement could not be performed in patients with obesity or ascites [29]. LS has been proven to be a good independent predictor of PHLF in HCC patients undergoing HR [20, 29], and patients with LS value≥15.7 kPa were at higher risk of PHLF [29]. At present, LS cannot be accurate enough to replace HVPG in determining the presence and severity of PHT [29], and its value of predicting the prognosis after HR for HCC needs to be further validated.

The model for end-stage liver disease (MELD) score, usually applied as a disease severity index for the waiting list for LT, has been proven to be an independent predictor of perioperative mortality, PHLF and long-term survival in patients with HCC and cirrhosis undergoing HR [30–32]. But the diagnostic capacity of MELD score was lower than that of HVPG [13]. HR was recommended in cirrhotic patients with HCC if the MELD score is less than 8, whereas other treatment strategies should be considered in patients with MELD score≥9 [32]. In patients with a MELD score of more than 10, the PHLF rate exceeded 15% [30].

Few studies has documented that combining these indexes to evaluate the liver function could be used to further select candidates with HCC undergoing HR [12], but various authors are agree with focusing on liver function [12, 22, 23, 30]. As in a current study, in patients with HVPG ≥10 mmHg but with a MELD score still below 10, HR can be safely operated in the condition of limited resection [12]. More studies providing a decision algorithm combining these indexes and further predicting the prognosis of HR are strongly awaited [12, 23].

Those surrogates of CSPH all have a quite wide “grey zone” in which their values can't rule out or rule in CSPH [33]. There are also other noninvasive of HVPG, such as imaging-derived strategies of CT angiography [34, 35] and MR imaging, which are not described in detail. Due to differences of definitions or evaluation methods of CSPH and different results, it is hard to get the conclusion that which kind of measurement is better [12], and additional data are still needed [16].

## VARIOUS FACTORS INFLUENCING THE IMPACT OF CSPH ON THE PROGNOSIS AFTER HR

In practice, lots of clinicians may be very interested in figuring out factors influencing the prognosis when HR is decided to be performed [22, 36]. Due to the advances in surgical techniques and preoperative care, perioperative mortalities during HR in HCC patients with CSPH could be reduced from 10.3% [21] to 6.7% [19, 22], which was also associated with the decrease of cirrhosis-related complications [15, 22]. Thanks to the advantages of mini invasion and reductions of HR-induced parenchymal injury and destruction of the collateral blood/lymphatic flow around the liver, laparoscopic HR could reduce the risk of PHLF and ascites and appear to be a safer option for HCC patients compared to open HR [37, 38]. Belli and colleagues confirmed the same impact of laparoscopic HR in HCC patients with preserved liver function and mild CSPH [39].

Although the EASL and AASLD guidelines recommend LT or RFA for patients with BCLC stage 0 or A HCC and CSPH who were regarded as “not ideal candidates” for HR [2, 3], the differences of the prognosis of various treatment modalities (e.g., LT or RFA) also have a strong appeal to clinicians [12, 16, 22, 23]. In a current study, *Roayaie* and *colleagues* reported that for those patients, HR was inferior to LT and RFA, which agreed with the guidelines, and was associated with better survival compared to embolization and other treatments [23]. But the result of the superiority of RFA over HR has to be interpreted carefully due to the consistent clinical difference between resected and ablated candidates [12]. But there are several studies demonstrating that RFS and OS were greater in patients with HCC and CSPH after HR than after RFA and HR was recommended [36, 37], although more frequent complications occurred [40]. In addition, the location and the extent of tumor still limit the application of LT and RFA, while HR is not [41]. For HCC patients with cirrhosis and CSPH whose disease extents were within the Milan criteria (single nodule≤5 cm, or up to three nodules none larger than 3 cm and no evidence of extra hepatic spread or vascular invasion), LT, regarded as the best modality, was highly recommended, the advantage of which is also treating the underlying liver disease [41]. HR can be considered the bridge to LT given the serious shortage of donor organs and long waiting times [12]. Compared to transcatheter arterial chemoembolization, HR also showed a better long-term survival in those “not ideal candidates” [21, 42].

## THE VALUE OF HVPG IN HCC PROGNOSIS AFTER LT

LT is a recommended curative treatment for early HCC in patients with cirrhosis, with the advantage of the treatments of not only tumors but also both the underlying liver disease [41, 43]. The fact that the donors' organs are of great shortage leads to prolonged waiting times which is one of the negative prognostic factor for HCC patients on the LT waiting list due to dropout and tumor progression [4]. Whether elevated HVPG belongs to one of the risk factors for HCC patients undergoing LT, such as tumor stages, high MELD score evaluating liver function and AFP level [44], need to be further explored, given that only one research, conducted by *Faitot* and *colleagues*, focused on that [43]. The results showed that CSPH diagnosed indirectly led to a higher risk of tumor progression in HCC patients which was the reason CSPH was regarded as a major risk factor of dropout [43]. It is interesting that patients with CSPH had lower efficiency of transcatheter arterial chemoembolization compared to those without CSPH, which might be explained by the changes of micro environmental including vascular endothelial growth factor pathways when existing CSPH [43, 45]. In addition, it is worth mentioning that the presence of CSPH had no significant impact on OS and the rate of recurrence of HCC patients after LT [43]. Further studies are warranted to validate the influence of HVPG on HCC prognosis after LT.

## THE VALUE OF HVPG IN THE PREDICTION OF HCC DEVELOPMENT

Several predictors of the development of HCC, such as viral etiology and AFP values, help to screen the high-risk population and identify HCC patients at early stages [8, 46, 47]. The influence of HVPG on the development of HCC in patients with compensated or decompensated cirrhosis has been investigated by only two studies [8, 48]. The first study showed that PHT is an independent predictor of the development of HCC in patients with compensated cirrhosis and PHT, and importantly, this association was independent from the degree of dysfunction and the duration of liver disease [8]. In addition, CSPH diagnosed directly was associated with an HCC incidence rate of 2.1% per year which was a 6-fold increase compared to non-CSPH and exceeded the cost-effective screening level of 1.5% [8, 49]. However, it should be noted that patients with varices were excluded and the results was still under debate mainly due to the selection bias and the set of endpoints[50]. Another study reported that in patients with decompensated alcoholic cirrhosis, HVPG greater than 15mmHg was an independent predictive factor for HCC development and was associated with a significantly shorter time for HCC development [48].

The mechanisms of the association between raised HVPG and the development of HCC are still unknown and might involve the process of fibrogenesis and angiogenesis [8, 51, 52]. Angiogenesis and structural abnormalities in the evolution of cirrhosis lead to the increase of hepatic vascular resistance and the reduction of sinusoidal perfusion, which contributes to the formation and the aggravation of PHT [51, 52]. And the value of HVPG reflects the degree of fibrogenesis and disruption of liver architecture to some extent [8]. Meanwhile, angiogenesis and fibrogenesis enhance the pathways of liver tumorigenesis [51]. Whether these associations can well explain the relationship between raised HVPG and HCC development need to be further validated. The predictive value of HVPG for the development of HCC also needs more exploration [50]. Since it is hard to routinely measure HVPG for the predictive purpose, noninvasive modalities should also be further investigated as a predictive tool.

## CONCLUSIONS

In conclusion, a raised HVPG diagnosed directly and CSPH measured indirectly both are negative prognostic factors for patients with HCC and cirrhosis, whereas that does not mean that the presence of CSPH (evaluated by indirect surrogate measurements) is an absolute contraindication to HR. Before deciding treatment modalities, it is necessary to take various factors into consideration, such as the extent of nodules and surgical techniques. It is needed to explore more about selecting indirect measurements of HVPG or algorithms combining several indexes and further predicting the prognosis of HR for patients with HCC and cirrhosis. Further studies providing more evidence on the value of HVPG in HCC prognosis after LT and prediction of HCC development are greatly awaited.

## Abbreviations

AASLD: American Association for the Study of Liver Diseases
BCLC: Barcelona Clinic Liver Cancer
CSPH: clinically significant portal hypertension
EASL: European Association for the Study of the Liver
EV: esophageal varices
HCC: hepatocellular carcinoma
HR: hepatic resection
HVPG: hepatic venous pressure gradient
LS: liver stiffness
LT: liver transplantation
PHLF: post-hepatectomy liver failure
PHT: portal hypertension
RFA: radiofrequency ablation
RFS: recurrence-free survival
MELD: the model for end-stage liver disease
OS: overall survival
